# Supplementary material for: Conserved hydrophilic checkpoints tune FocA-mediated formate:H+ symport
Source: Nat Commun. 2025 Oct 27;16:9476. doi: 10.1038/s41467-025-65159-3 (PMC12559229; doi:10.1038/s41467-025-65159-3)
Supplement: Supplementary file 1 — Supplementary Information [file 41467_2025_65159_MOESM1_ESM.pdf]

SUPPLEMENTARY MATERIAL

Conserved hydrophilic checkpoints tune FocA-mediated formate:H⁺ symport

Christian Tüting[1,2#], Kevin Janson[1,2#], Michelle Kammel[3#], Christian Ihling[4,5], Jana Lorenz[4,5], Fotis L. Kyrilis[1,6], Farzad Hamdi[1,2], Christopher Erdmann[3], Andrea Sinz[4,5], R. Gary Sawers[3]*, Panagiotis L. Kastritis[1,2,6,7]*

[1]Interdisciplinary Research Center HALOmem, Charles Tanford Protein Center, Martin Luther University Halle-Wittenberg, Kurt-Mothes-Straße 3a, Halle (Saale), Germany

[2]Institute of Biochemistry and Biotechnology, Martin Luther University Halle-Wittenberg, Kurt-Mothes-Straße 3, Halle (Saale), Germany

[3]Institute of Biology/ Microbiology, Martin Luther University Halle-Wittenberg, Kurt-Mothes-Str. 3, 06120 Halle (Saale), Germany

[4]Department of Pharmaceutical Chemistry & Bioanalytics, Institute of Pharmacy, Martin Luther University Halle-Wittenberg, Kurt-Mothes-Str. 3, 06120 Halle (Saale), Germany

[5]Center for Structural Mass Spectrometry, Martin-Luther University Halle-Wittenberg, Kurt-Mothes-Str. 3, 06120, Halle (Saale), Germany

[6]Institute of Chemical Biology, National Hellenic Research Foundation, 11635 Athens, Greece

[7]Biozentrum, Martin Luther University Halle-Wittenberg, Weinbergweg 22, Halle (Saale), Germany

[#]C.T., K.J. and M.K. contributed equally to this work.

*address correspondence to gary.sawers@mikrobiologie.uni-halle.de or panagiotis.kastritis@bct.uni-halle.de

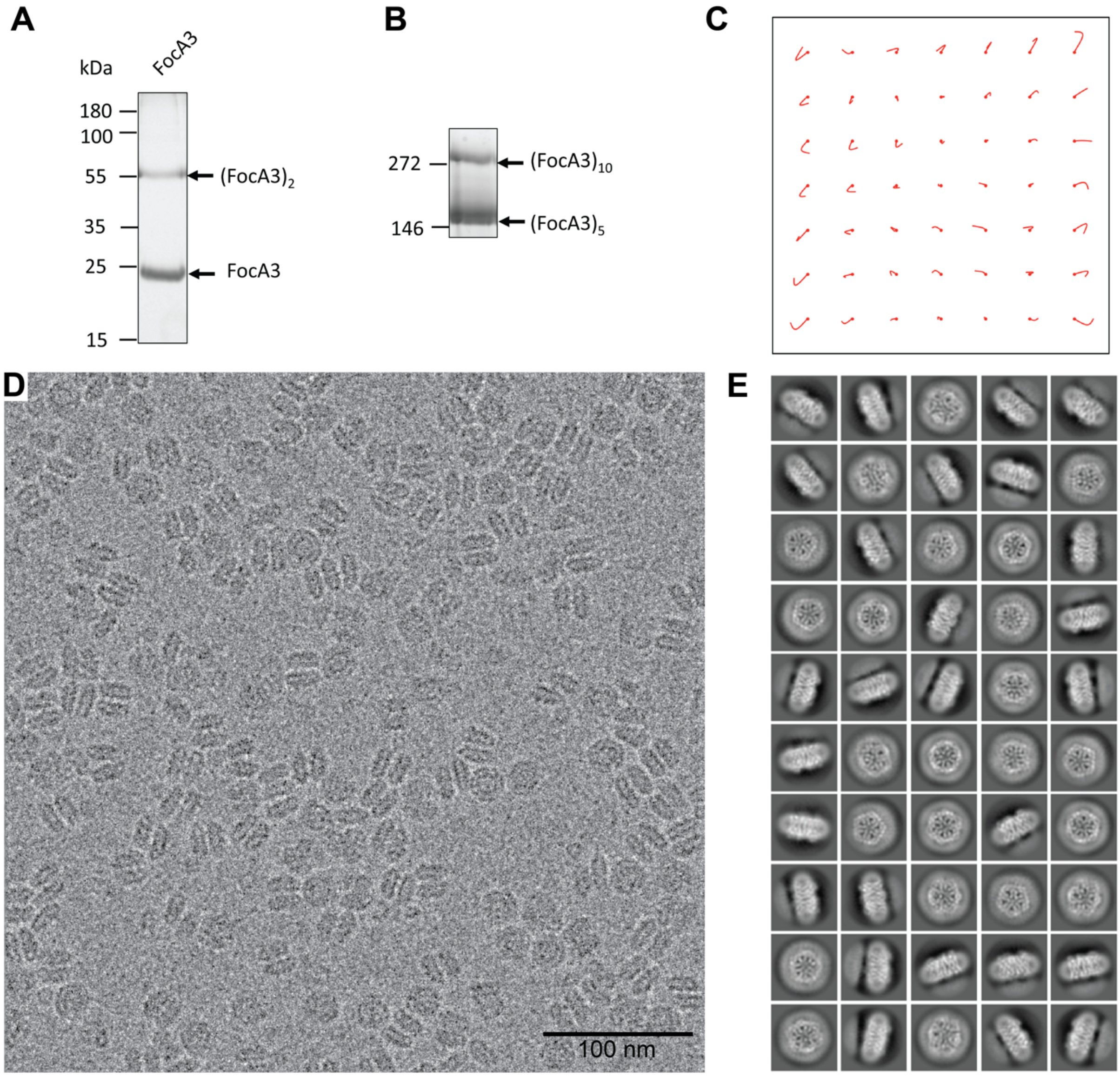

Supplementary Figure 1. Synthesis and membrane-integrity of FocA (H209) are not altered compared to native FocA and FocA3 forms. (A) An aliquot (2 µg) of purified FocA (FocA3) was separated in a denaturing 12.5% (w/v) polyacrylamide gel and subsequently silver-stained (FocA3 signifies the recombinant protein with a *C*-terminal strepII-tag). (B) An aliquot (25 µg) of purified FocA3 was analyzed by Blue-native (BN) gel electrophoresis using a 4 – 16% (w/v) polyacrylamide gradient. The migration positions of the molecular mass markers, in the case of denaturing SDS-PAGE PageRuler Prestained Protein Ladder (Thermo Fisher Scientific), and for the BN-PAGE a Serva marker including jack bean urease (272 kDa) and porcine lactate dehydrogenase (146 kDa) are indicated in kDa on the left of the figure. The arrows indicate the monomer form of FocA3, the dimer $(FocA3)_2$, the pentamer $(FocA3)_5$ and the decameric form of the FocA protein $(FocA3)_{10}$. (C) Cryo-electron microscopy micrograph as an example of the vitrified sample of native, wild-type FocA. (D) Example of a motion correction image showing limited drift during movie acquisition in the Glacios 200 kV. (E) Example of 2D class averages from the wild-type FocA dataset, showing the different orientations of the pentameric complex. Uncropped gels are presented in Supplementary Figure 22.

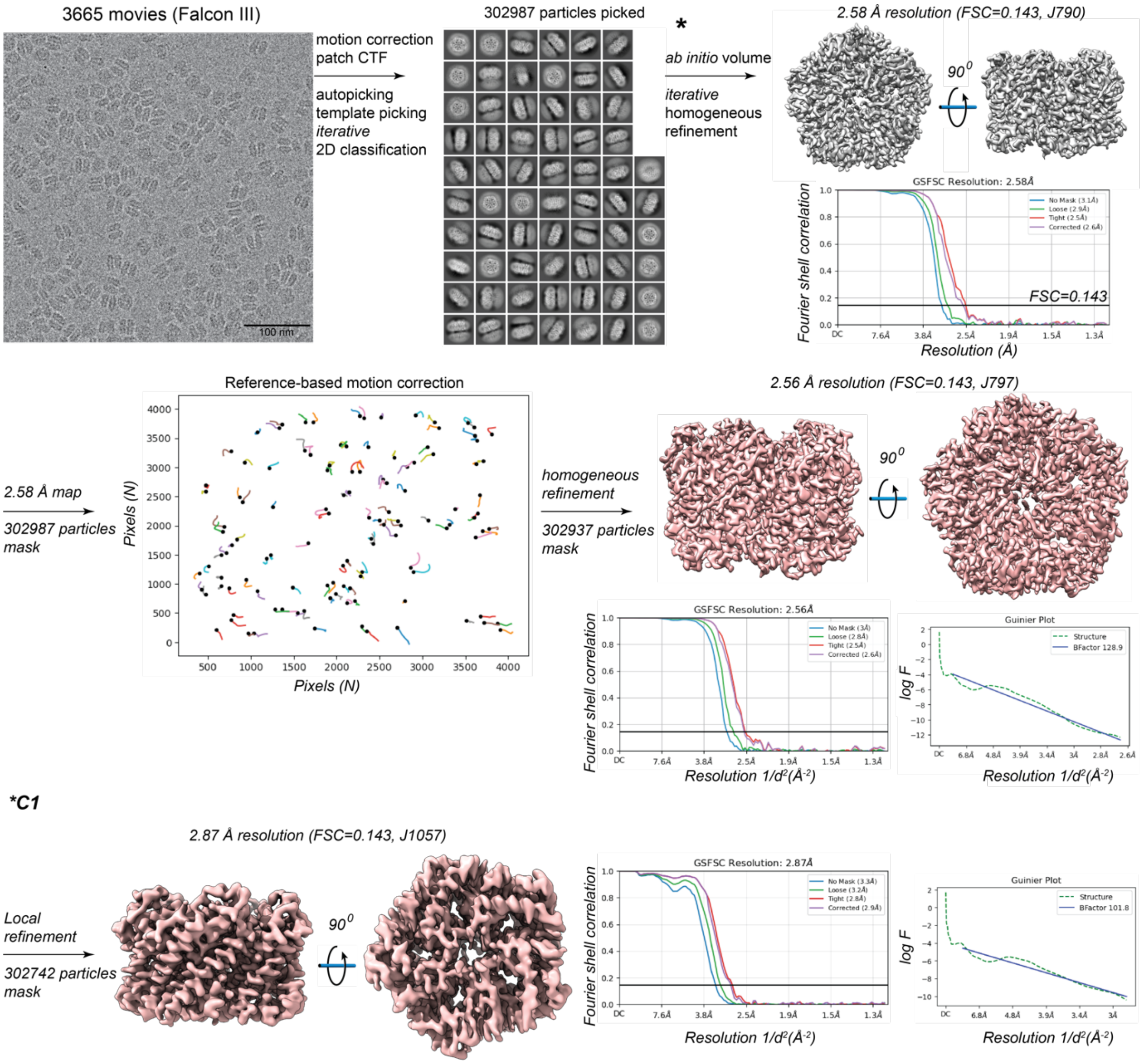

Supplementary Figure 2. Image processing strategy for analyzing the native FocA cryo-EM structure using cryoSPARC 4.4. Original micrographs were motion-corrected and CTF was estimated. FocA particles were picked using template picker, and iteratively classified (2D), followed by a 3D reconstruction (*ab initio* and homogenous refinement). To increase resolution, per-particle motion-correction was performed (Reference-based motion correction), followed by 3D reconstruction, resulting in the final map with a resolution of 2.56 Å. For asymmetric reconstruction, a Local Refinement without imposing symmetry (C1) was performed, resulting in a resolution of 2.87 Å.

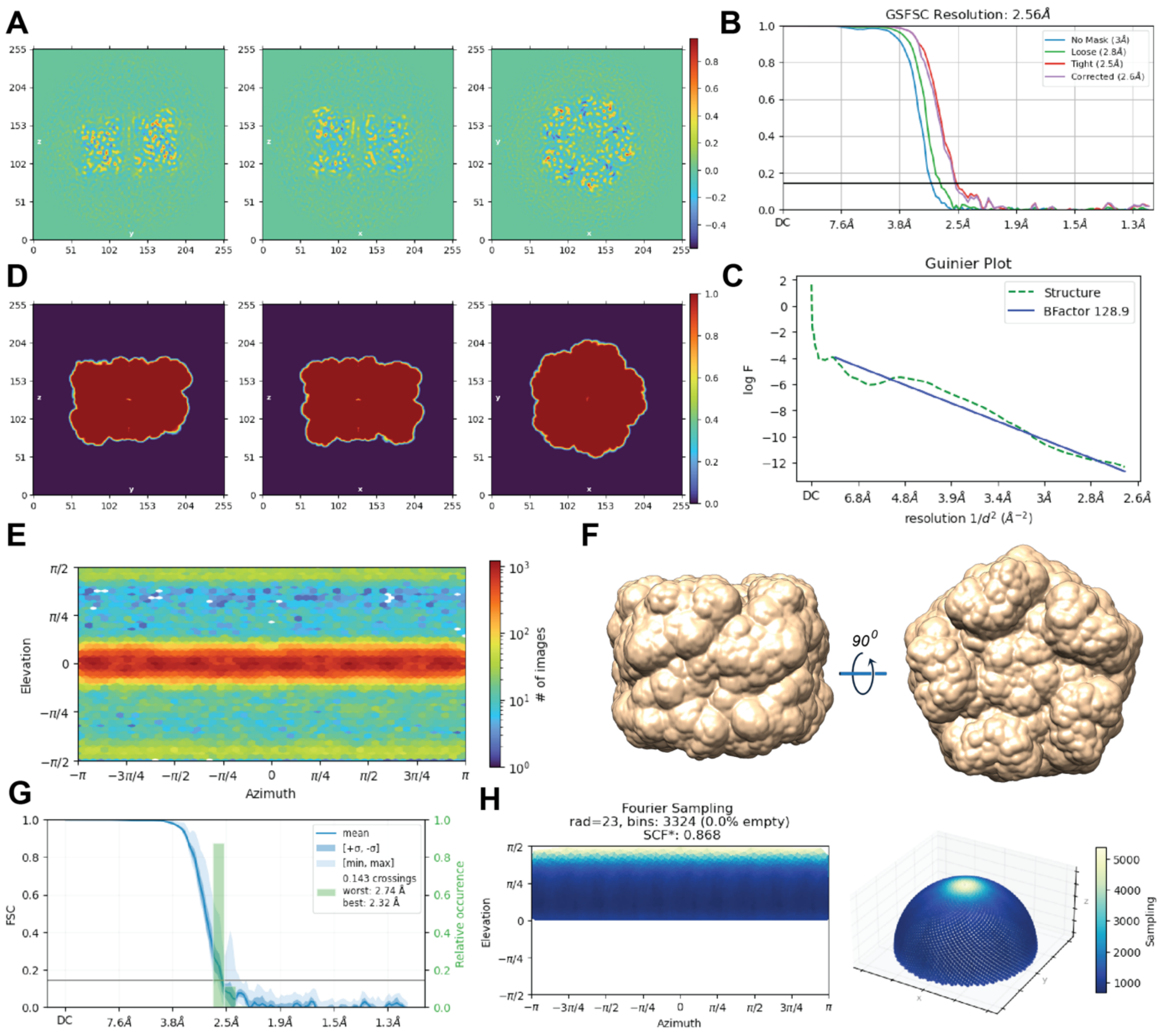

Supplementary Figure 3. (A) Slices of the final FocA cryo-EM map. (B) FSC plot indicating resolution of FocA at 0.143 threshold. (C) Guinier Plot, calculating the B-factor of the FocA cryo-EM map. (D) Slices of the mask used to calculate corrected FSC resolution. (E) Enrichment of particles in the final cryo-EM map, indicating that all views are covered. (F) Volume of the applied mask. (G) Calculation of 3D FSC, which shows minimum deviation across resolution values, indicating good coverage of views in the final reconstruction. (H) Calculation of SCF.

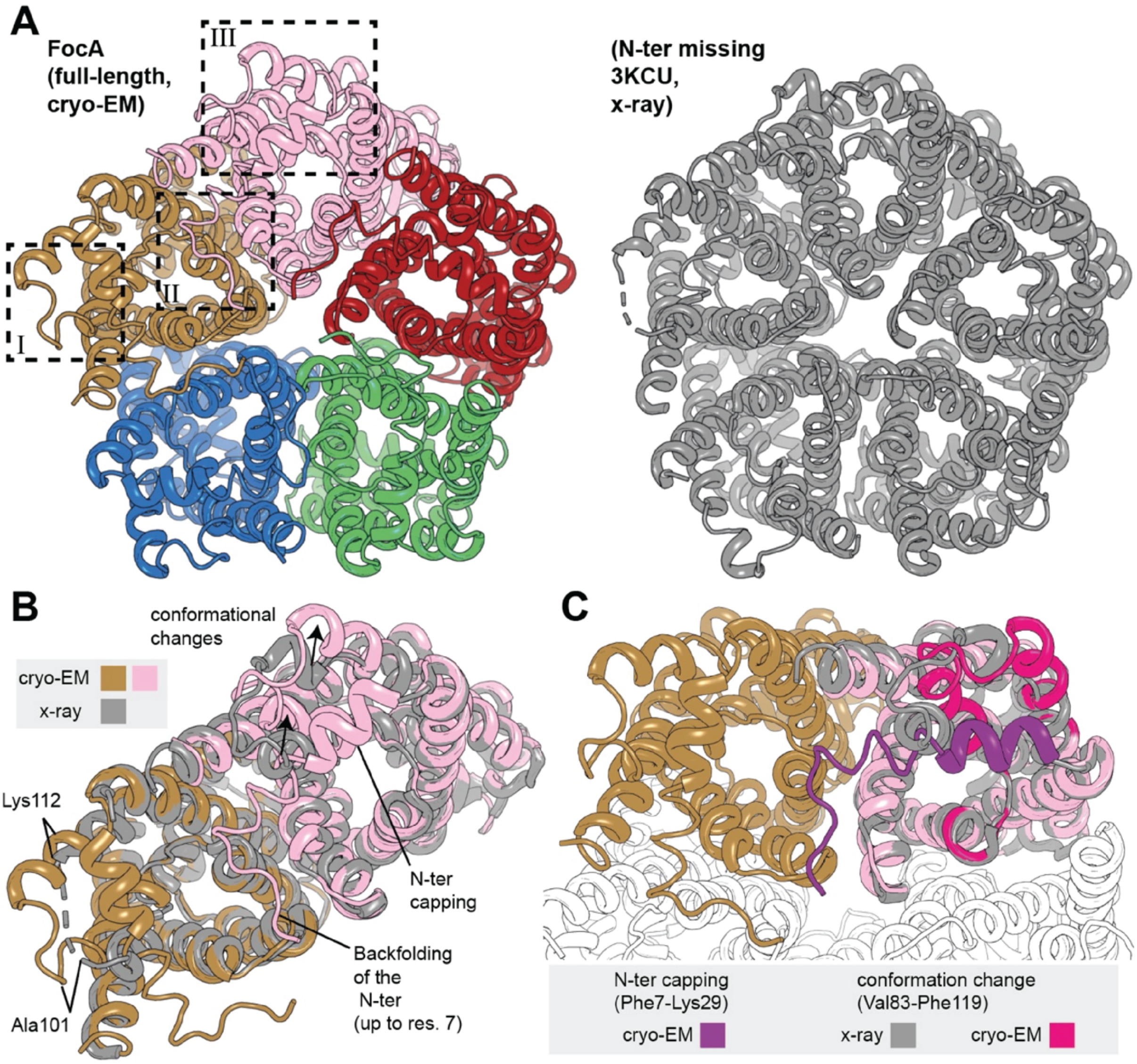

Supplementary Figure 4. Cryo-EM model of FocA channel. The structure is compared with the previously resolved FocA channel from *E. coli* lacking the *N*-terminal helix (residues 1-21). **A**. The overall architecture shows both structures are highly similar, and are composed of 5 protomers. Localized changes are highlighted inside the dotted squares and labelled I, II and III. **B**. Annotation of conformational changes observed for the cryo-EM-resolved structure compared to the X-ray-derived counterpart (PDB ID 3KCU). **C**. *N*-terminal capping of the cytoplasmic portion of the channel and the structural changes induced by the presence of this structural element (V83-F119 segment) are highlighted.

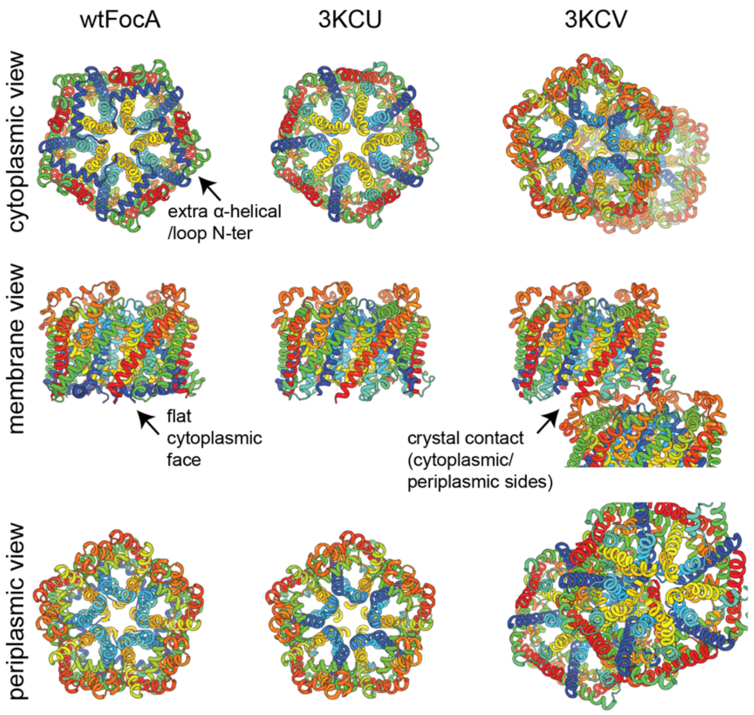

Supplementary Figure 5. **Comparative analysis regarding the overall structure across all resolved FocA molecules.** The three views (cytoplasmic, membrane, periplasmic) of all resolved FocA structures are shown. PDB IDs are noted above each structure. Each chain is colored in rainbow (Blue to red from *N*- to *C*-terminus) and shown in cartoon form. Crystal contacts are shown, if applicable.

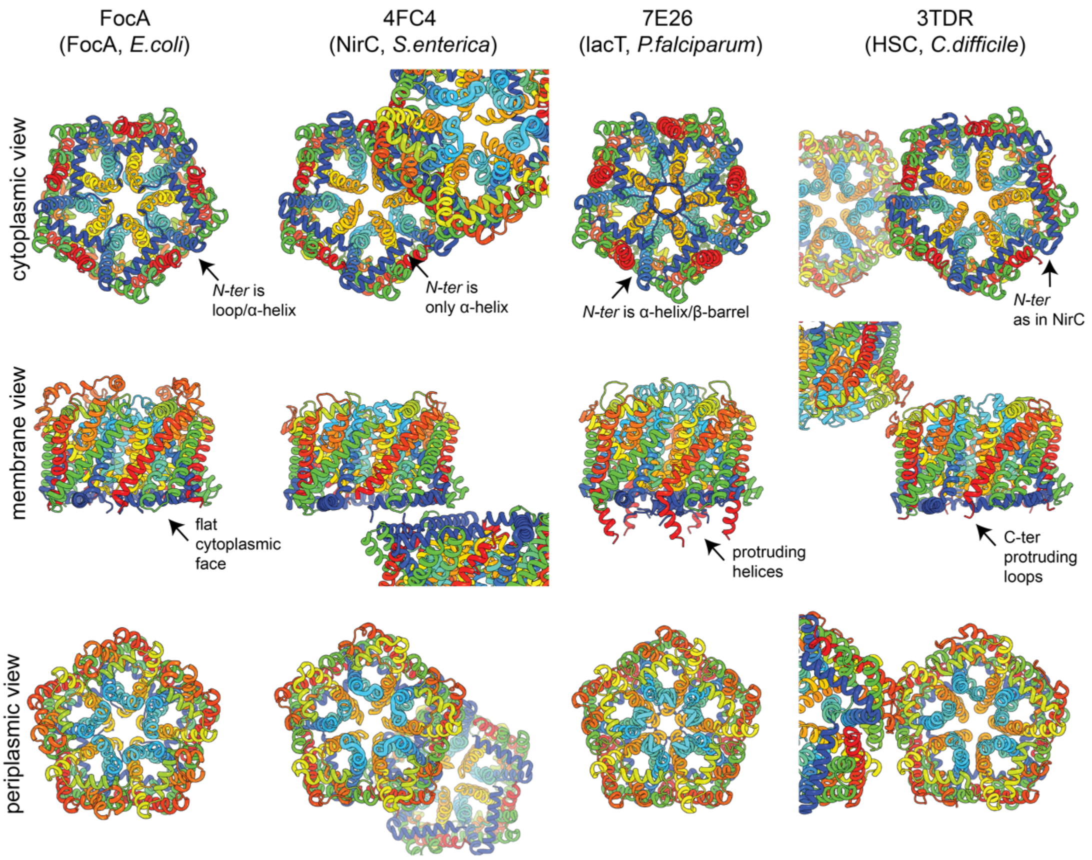

Supplementary Figure 6. Comparative analysis regarding the overall structure across all resolved FNT transporters. The three views (cytoplasmic, membrane, periplasmic) of all selected FNT structures are shown. PDB IDs, genes and organisms are noted above each structure. Each chain is colored in rainbow (Blue to red from *N*- to *C*-terminus) and shown in cartoon form. Crystal contacts are shown, if applicable.

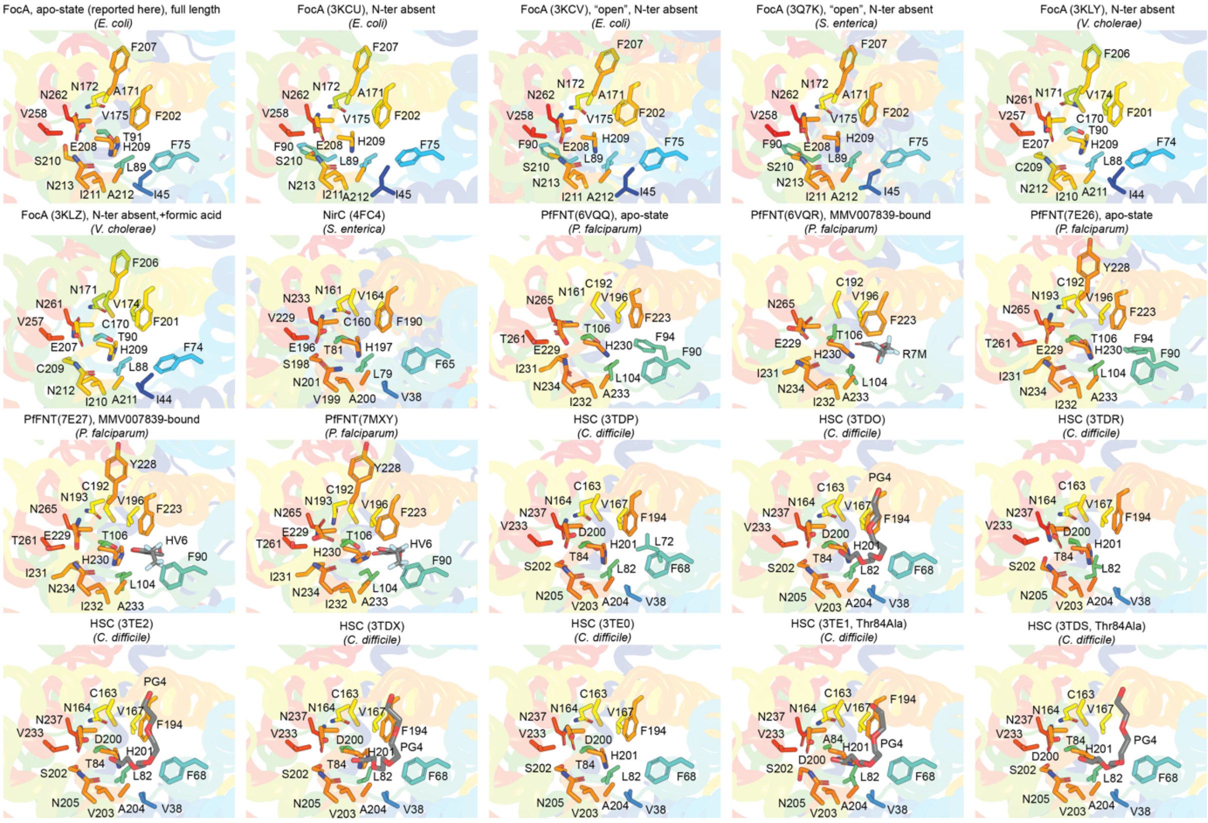

Supplementary Figure 7. Conservation of the histidine residue across resolved structures of FNT transporters. Not only the side-chain conformation of the histidine is conserved, but also amino acid residues in the pore within its proximity (threshold is 5 Å).

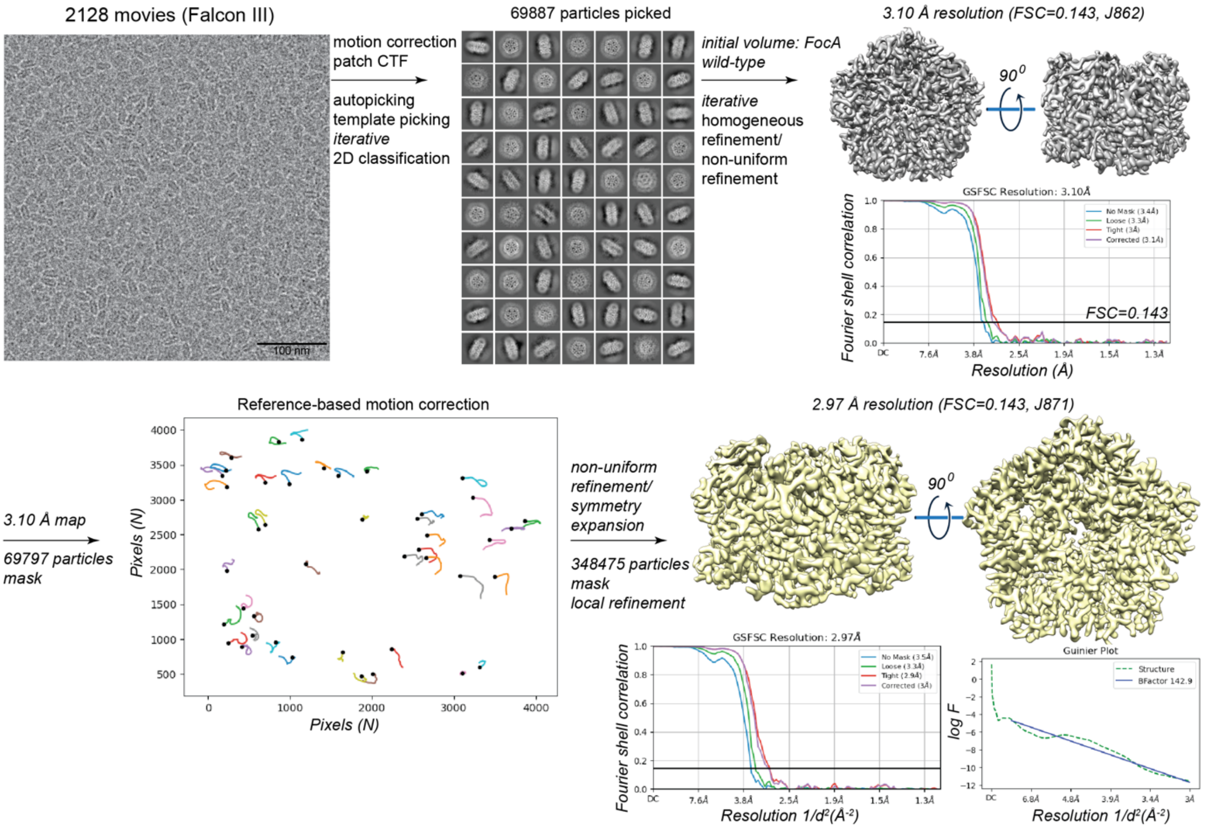

Supplementary Figure 8. Image processing strategy for analyzing the FocA-H209N cryo-EM structure using cryoSPARC 4.4. Original micrographs were motion-corrected and CTF was estimated. FocA particles were picked using template picker, and iteratively classified (2D), followed by a 3D reconstruction (*ab initio* and homogenous refinement). To increase resolution, per-particle motion correction was performed (Reference-based motion correction), followed by symmetry expansion and 3D reconstruction, resulting in the final map with a resolution of 2.97 Å.

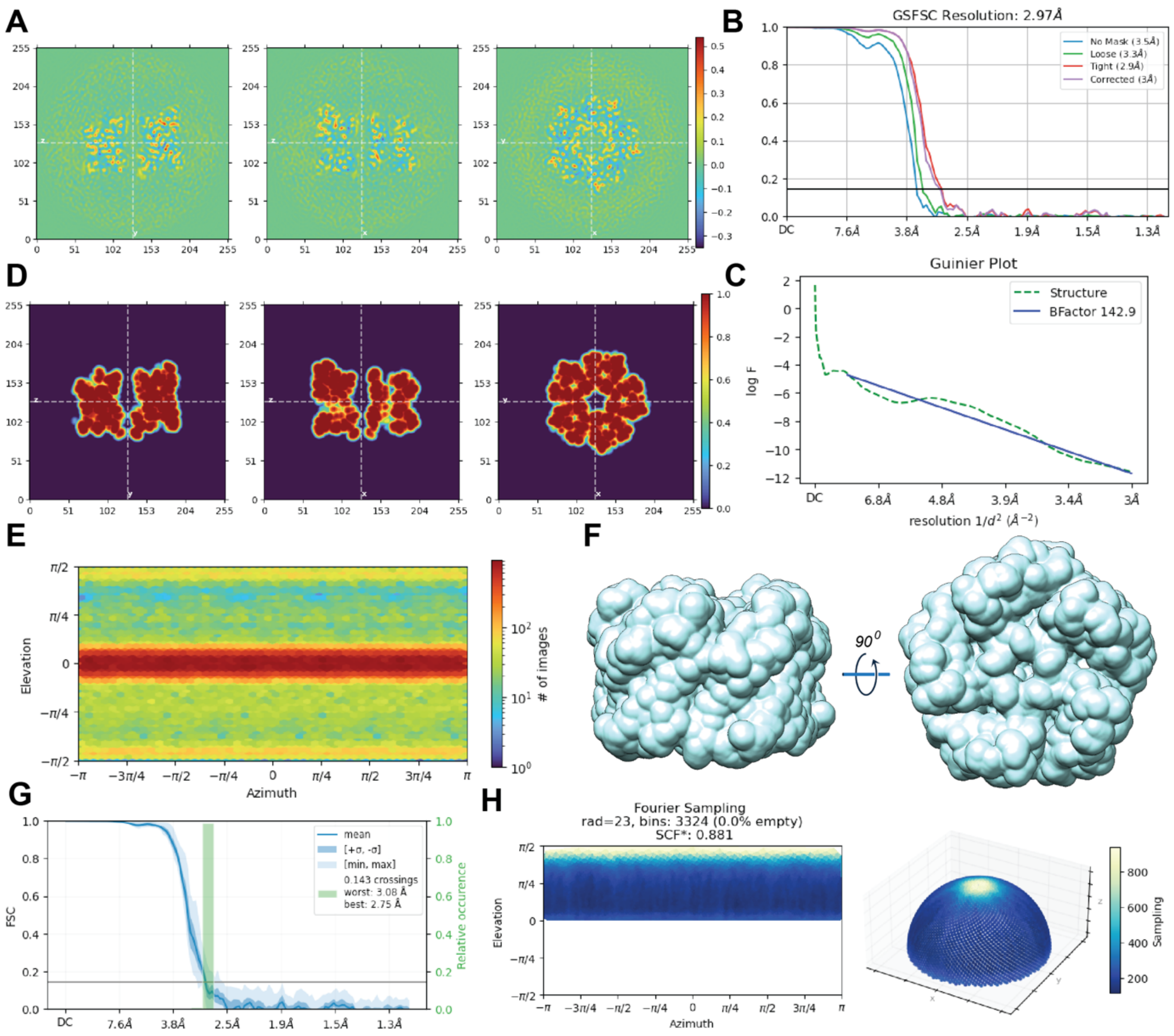

Supplementary Figure 9. (A) Slices of the final FocA-H209N cryo-EM map. (B) FSC plot indicating resolution of FocA-H209N at 0.143 threshold. (C) Guinier Plot, calculating the B-factor of the FocA cryo-EM map. (D) Slices of the mask used to calculate corrected FSC resolution. (E) Enrichment of particles in the final cryo-EM map, indicating that all views are covered. (F) Volume of the applied mask. (G) Calculation of 3D FSC, which shows minimum deviation across resolution values, indicating good coverage of views in the final reconstruction. (H) Calculation of SCF.

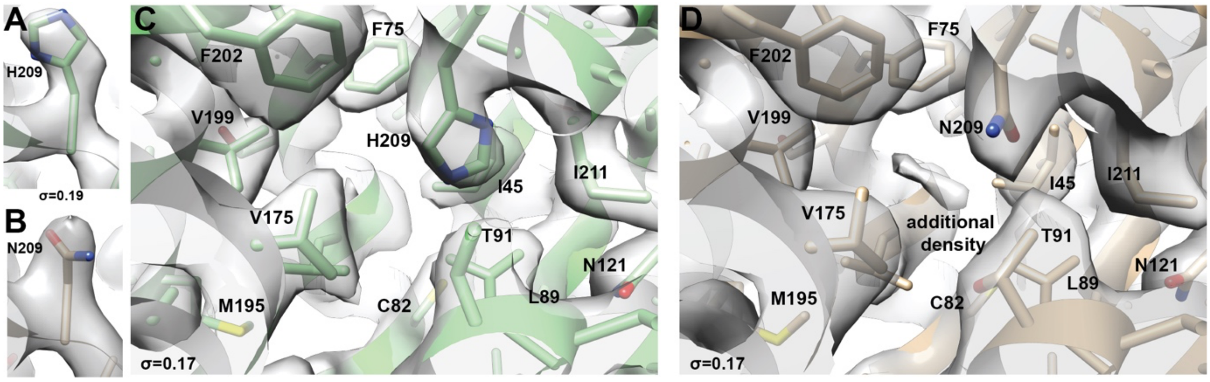

Supplementary Figure 10. Comparison of densities at the same sigma map threshold. (A) Refinement of H209 in the native FocA cryo-EM map. (B) Refinement of the N209 in the FocA-H209N cryo-EM map. (C) View of the FocA pore, and the residues surrounding the H209 residue. (D) Same as (C), but for the H209N variant; an additional density is apparent proximal to the exchanged residue (N209).

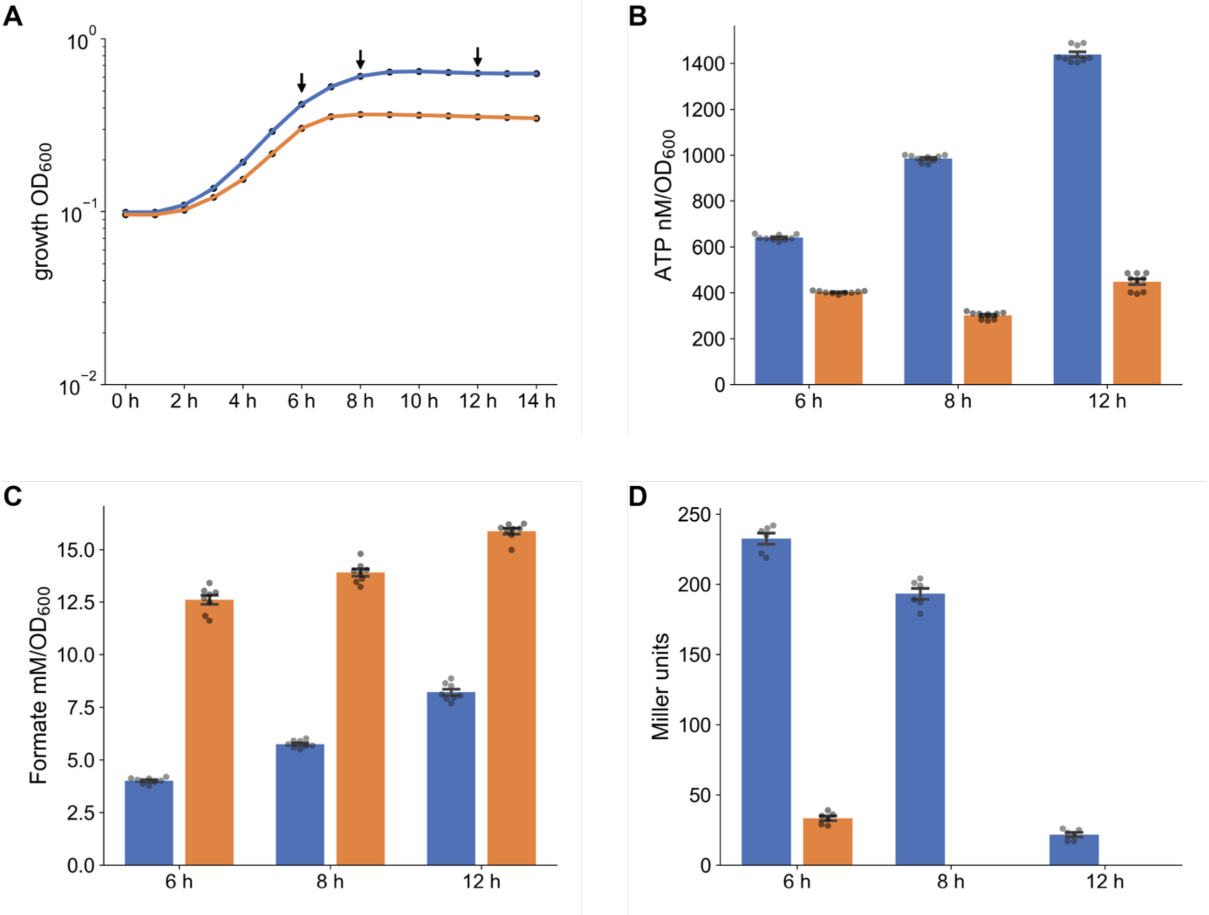

Supplementary Figure 11. Fermentative growth and ATP levels are restricted in the formate-effluxing *E. coli* mutant synthesizing FocA-N209. Anaerobic growth in M9-glucose minimal medium. (A). The vertical arrows highlight where samples were taken for determination of intracellular ATP concentration (B), extracellular formate concentration (C), and intracellular formate-dependent β-galactosidase enzyme activity (D). All experiments were done with three biological replicates, with each biological replicate performed in duplicate. Data are presented as mean values +/- SEM.

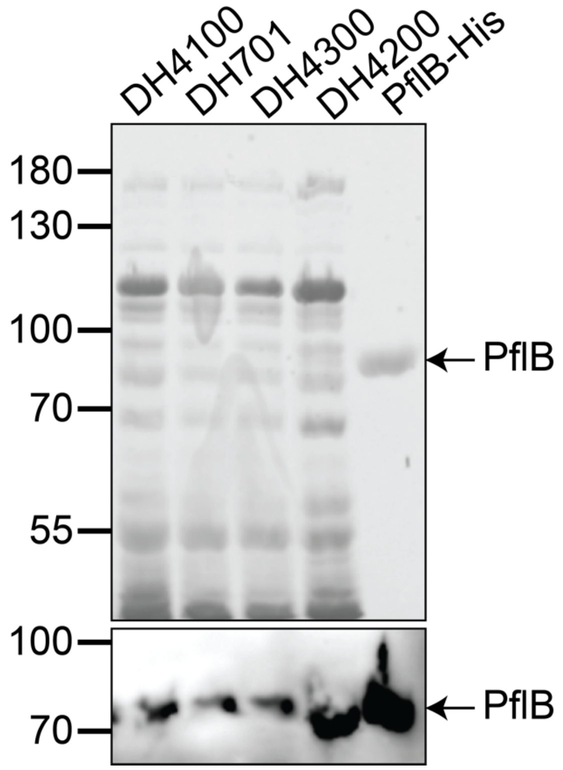

Supplementary Figure 12. PflB is stably synthesized in strains carrying mutations in *focA*. Upper panel: SDS-PAGE (12.5% w/v acrylamide) analysis of polypeptides present in crude extracts (25 μg protein) of the indicated strains. DH4100 (wild-type FocA); DH701 (no FocA); DH4300 (synthesizes FocA-T91A); DH4200 (synthesizes FocA-H209N); purified N-terminally His-tagged PflB (1 μg protein). The gel was silver-stained (see Methods). Lower panel: western blot of the same samples separated in a parallel SDS-PAGE and PflB was identified with PflB antiserum (diluted 1:3000). This antiserum was raised in rabbits and contain antibodies that recognize purified PflB. Uncropped blots are presented in Supplementary Figure 23.

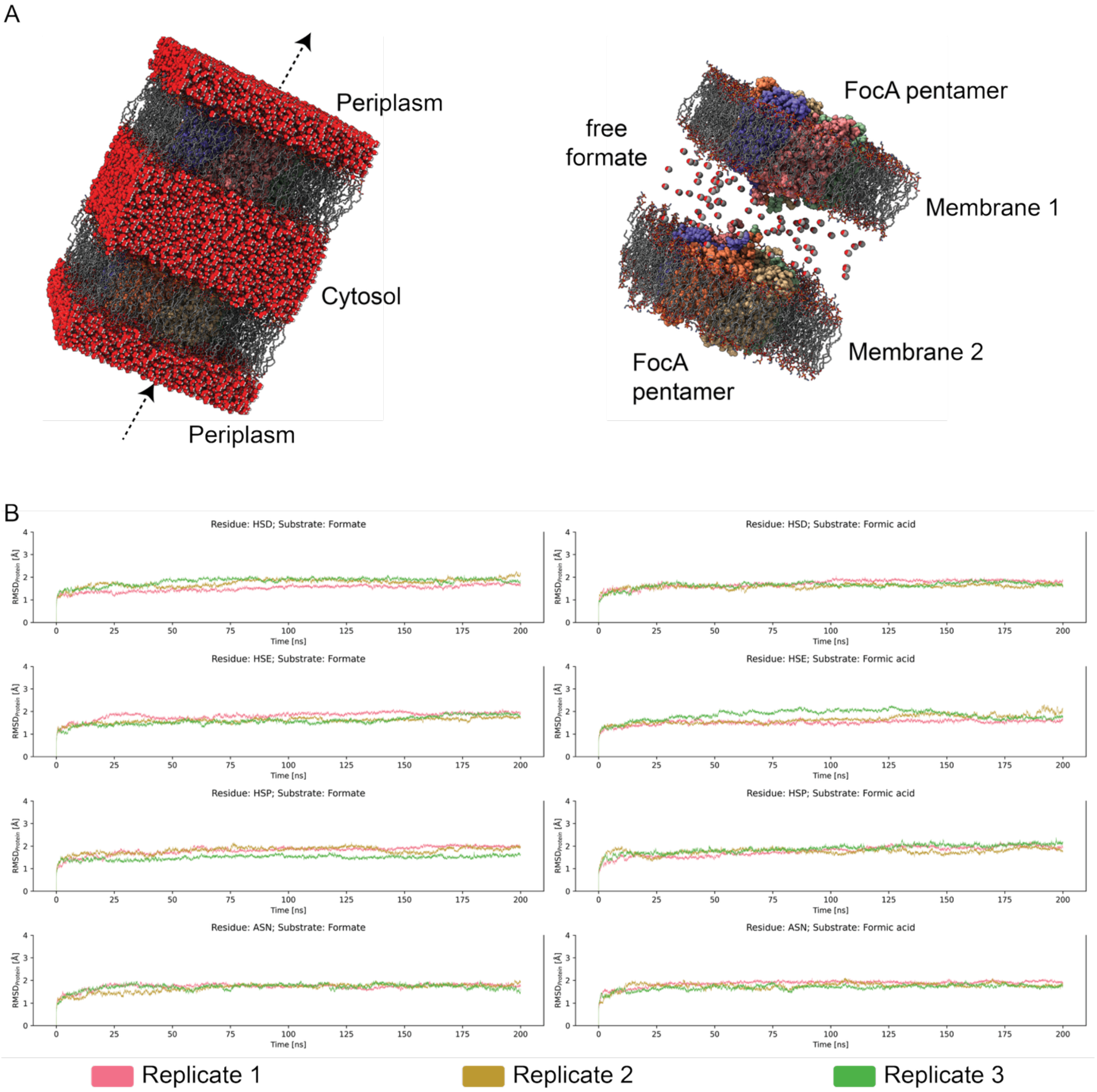

Supplementary Figure 13. MD simulation of FocA. (A) The simulation box. The simulation was performed with active periodicity, meaning that the edges are connected via the box boundaries. This is indicated by the dotted arrow line for the z-periodicity but also applies to the x and y axes. Hereby, enclosed cytosolic and periplasmatic compartments are formed. Each membrane system contains one FocA pentamer, and the cytosolic volume is saturated with 200 mM free formate. (B) RMSD values of the protein backbone during the 200 ns simulations. The protein is stable over the time-course of the simulation.

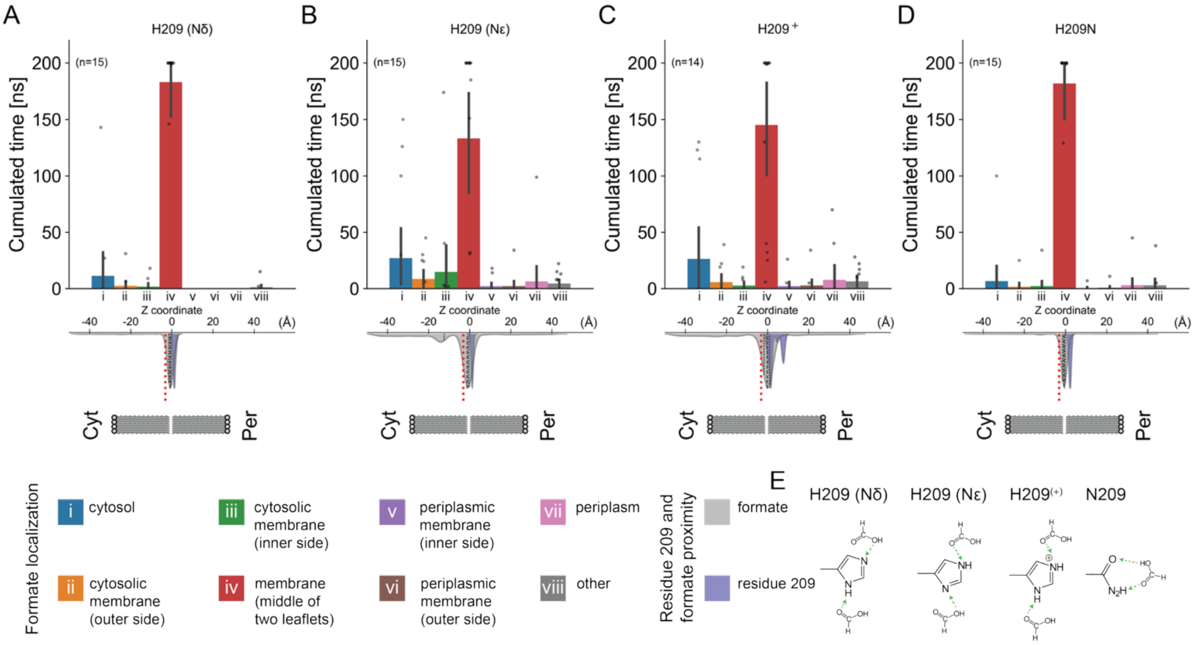

Supplementary Figure 14. Molecular dynamics simulations show formic acid hydrogen-bonding dependent retention. (A-D) Bar plots of formic acid localization, and violin plots for substrate (grey) and residue 209 (purple). (A) For the H209 Nδ tautomer, formic acid is attracted to H209 and retained due to hydrogen bonding. (B) same as (A), but slightly reduced. (C) $H209^{+}$ preferentially retains formate **(Fig. 3C)** as compared to formic acid, which follows similar preferences as in (B). (D) H209N strongly attracts formic acid due to complementary hydrogen bonding (see (E)). Formic acid can also displace bidirectionally. Dotted lines indicate the 25th and 75th quartiles, and the dashed line the data median. Substrate localization for formate is shown in **Fig. 3**, per-replicate violin plots are shown in **Fig. S15**. The red dashed line indicates the starting point of the substrate. Statistical details regarding the analysis are described in Methods; "Other" is any position calculated not falling within the 7 other categories. (E) Site-specific hydrogen-bonding propensities. Neutral histidine is in a slow tautomeric equilibrium.

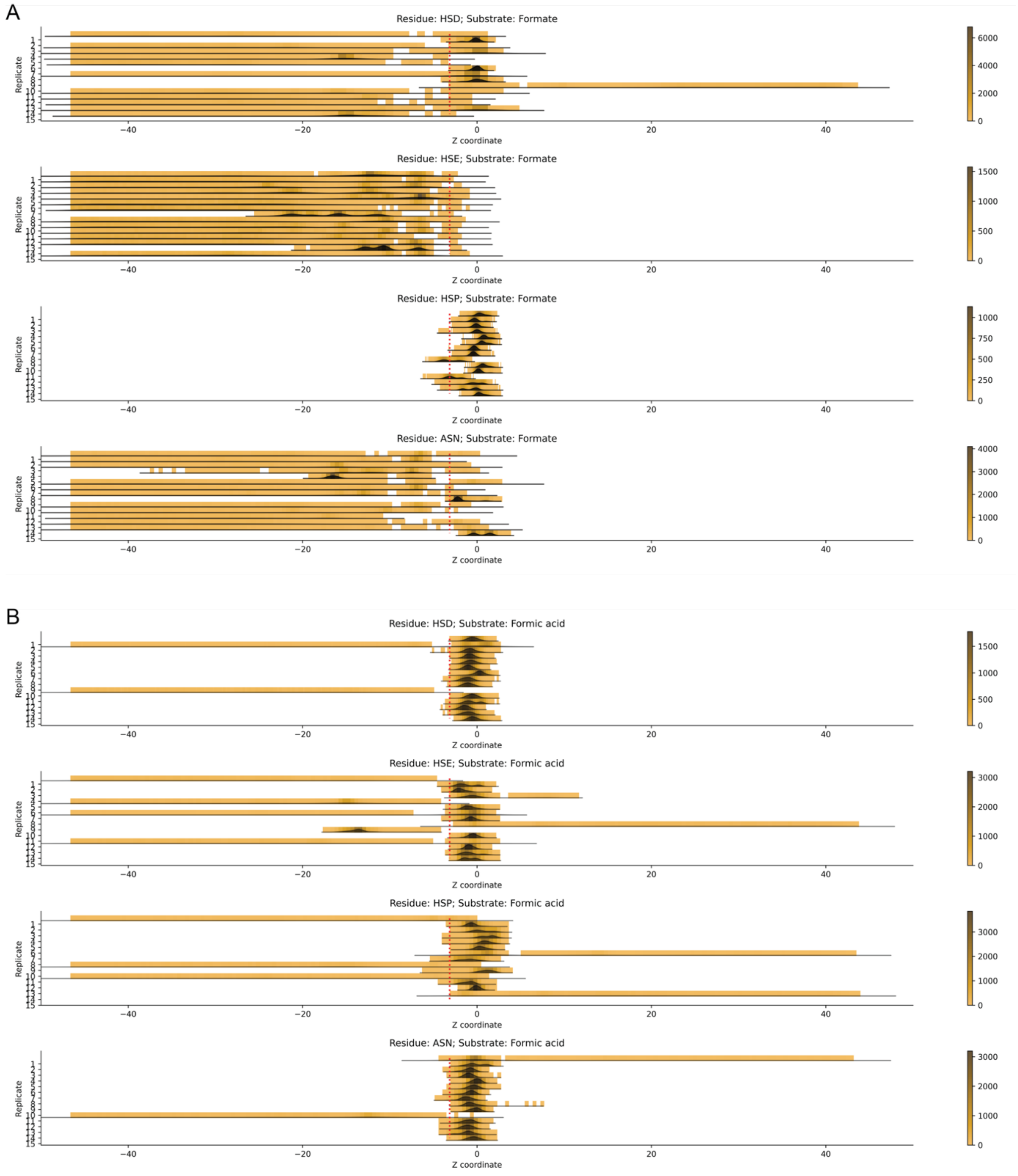

Supplementary Figure 15. Per-replicate histogram (yellow) and violin plots (black) for analyzing the location of the bound substrate; the analysis considers every frame (frame interval is 20 ps). Histograms were generated with a bin size of 50, covering ~2 Å within each bin.

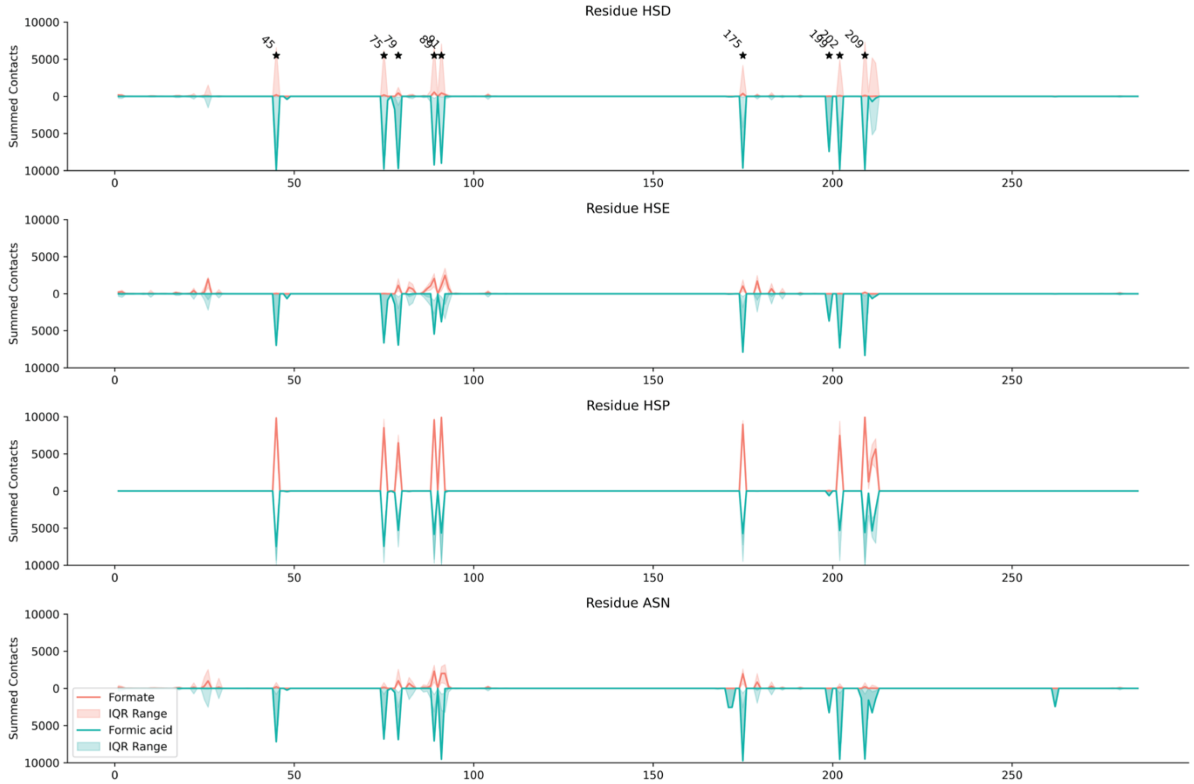

Supplementary Figure 16. Contacts of the substrate within the channel. A distance of below 4 Å was considered as contact distance, and counted contacts were condensed to the residue level, meaning that multiple atom-atom contacts concerning the same residue were considered as a single occurrence so as not to bias towards a residue. Residues in contact at the beginning of the simulation are indicated by an *, pointing to a stable interaction of the substrates with FocA at the exact location of the cryo-EM H209N variant map. In addition, more residues show contacts, such as those in-between residues 175-199, residues 210-211, and N-terminal residues. Total protein contacts across frames per replicate (n=15 per condition) were statistically analyzed (median, inter-quartile (IQR, 25th and 75th percentile)).

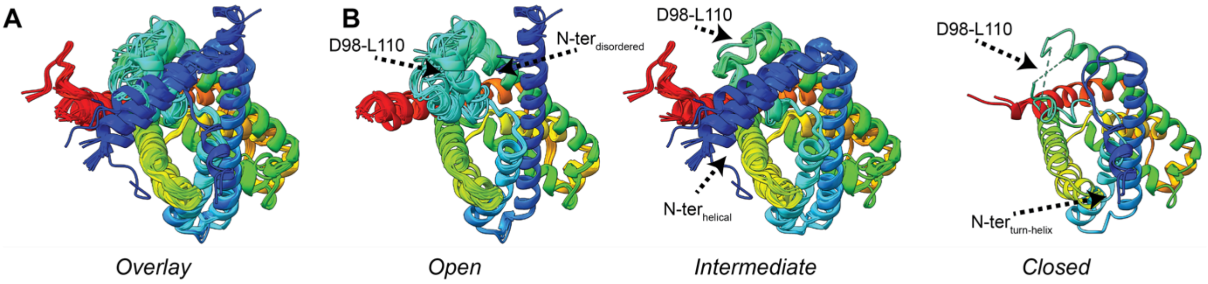

Supplementary Figure 17. Comparison of structurally resolved *N*-terminal helix on FNTs. Structures were taken from the following PDB IDs: 3KCU[1], 3KCV[1], 3KLY[2], 3KLZ[2], 3Q7K[3], 3TDO[4], 3TDP[4], 3TDR[4], 3TDS[4], 3TDX[4], 3TE0[4], 3TE1[4], 3TE2[4], 4FC4[5], as well as our cryo-EM reconstruction. Each structure was downloaded as a biological assembly, and 'split' by chains. Each chain was aligned to our cryo-EM structures. The overlay of all structures is shown in (A). (B) Sub-classification of different states of FNTs, based on Lü. et al.[3]. In the open state, protomers from 5 structures (from 3KLY, 3KLZ, 3KCU, 3KCV, 3Q7K), in the intermediate state, which is also re-ensembled in our cryo-EM structure, protomers from 10 structures (from 3TDO, 3TE1, 3TDP, 3TE0, 4FC4, 3TDS, 3TDR, 3TDX, 3TE2, 3Q7K), and in the closed state protomers from only 1 structure (3Q7K). Based on the alignments it is clear that in the "intermediate" state, the *N*-terminal helix stabilizes the D98-L110 loop in a distinct conformation. Only if the *N*-terminal helix is either disordered (open state), or in a different conformation (closed state), and thereby not in a stable interaction with the loop region, are clear conformational changes are observed.

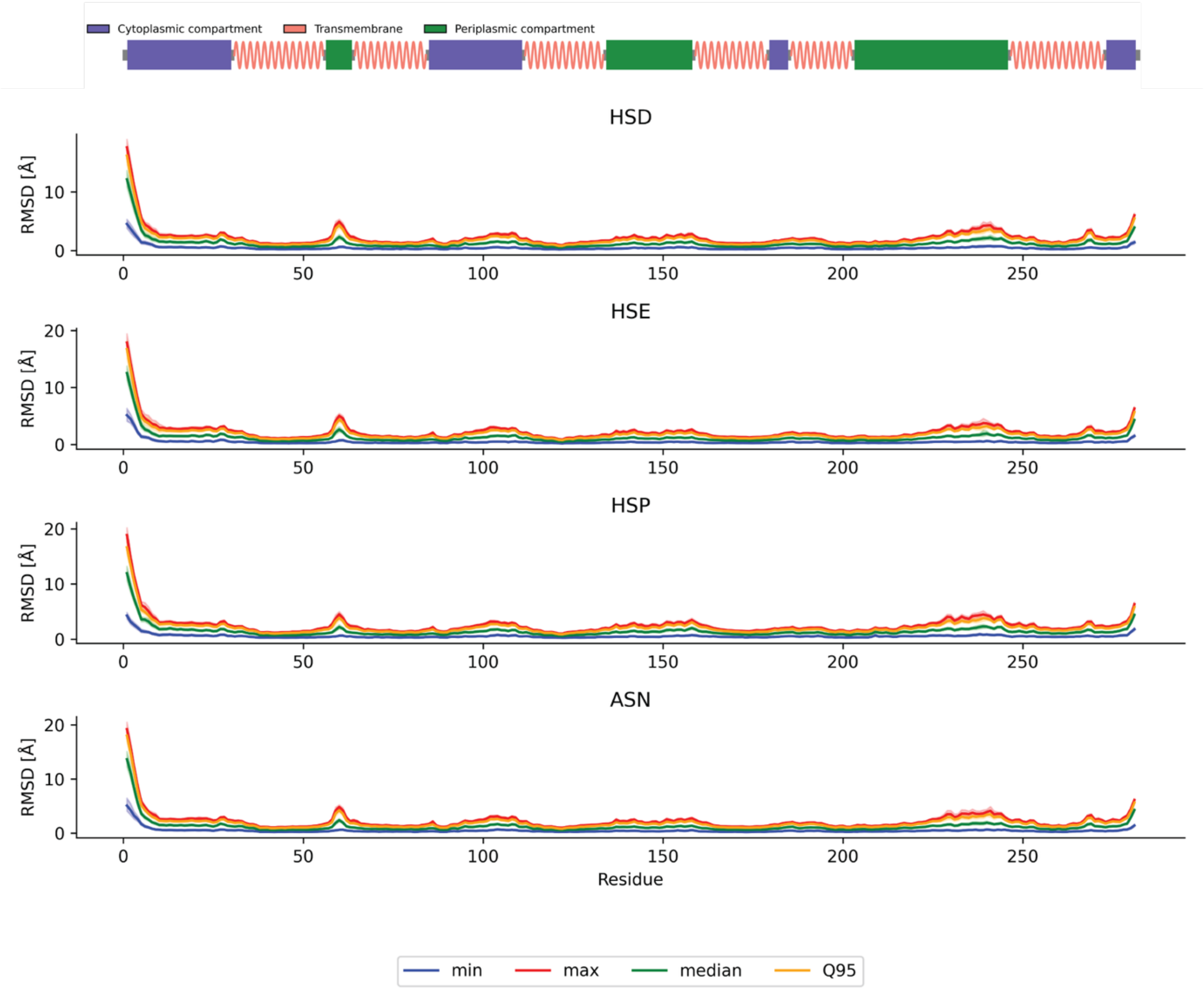

Supplementary Figure 18. Per-residue RMSDs throughout the MD simulations. The upper panel is based on FocA UniProt annotation. Each plot show the residue number on the X-axis. The per-residue RMSD, compared to the first frame was calculated per protomer (formic acid/formate simulations are merged, therefore, 10 protomers are considered per simulation, 3 triplicates; n=30), and median and 95% quantile over every 250th frame (total 1200 datapoints per residue) calculated using the python package pandas. Transmembrane α-helices show only small fluctuations, whereas periplasmic regions are more flexible, corroborating the 3D variability analysis from the cryo-EM data **(Fig. 4)**. Labels are based on the CHARMM classification of each residue: HSD: Nδ; HSE: Nε; and HSP: Nδ and Nε protonation.

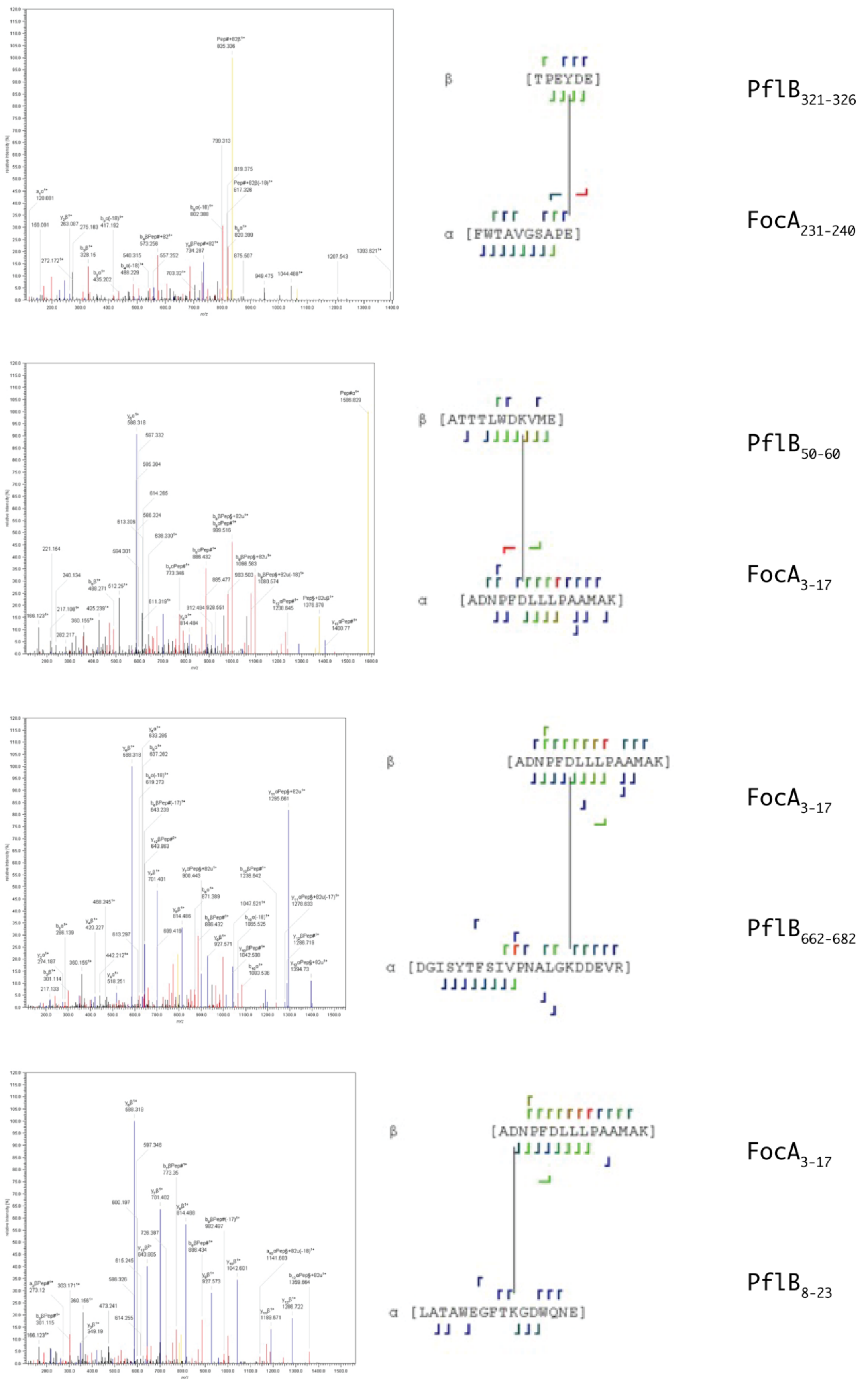

Supplementary Figure 19. Exemplary MS/MS spectra of cross-linked peptides. Annotated spectra are exported from MeroX. Peptide location and cross-linked residues are

indicated. Please note, that in the FocA3–17 peptide, the cross-link site cannot be unambiguously assigned between D4 and D8. This is because the ester formed during SDA cross-linking fragments before the peptide backbone, leaving the peptide unmodified. For illustration, MeroX displays one of the equally possible sites arbitrarily.

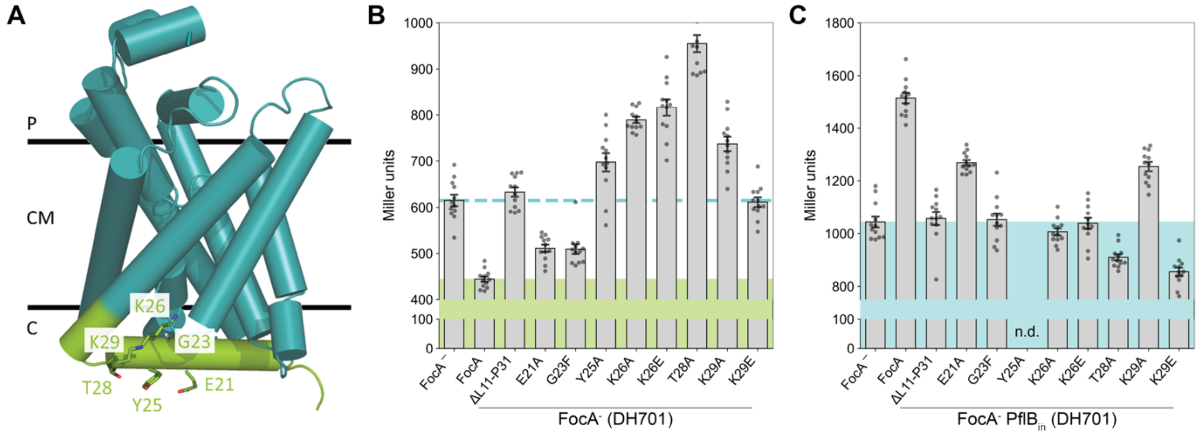

Supplementary Figure 20. Formate translocation by FocA relies on key amino acids in the *N*-terminal domain. A. In the schematic representation of a FocA protomer, the amino acids in the distal region of the *N*-terminus chosen for substitution are highlighted. FocA is shown in cartoon representation with α-helices as cylinders and its cytoplasmically-oriented *N*-terminal helix (residues P12-K29), which runs parallel with membrane, is colored green. The position of FocA within the cytoplasmic membrane (CM) is indicated and the boundaries of the cytoplasm (C) and periplasm (P) are shown. **B** Formate translocation was investigated in the *focA* mutant ($FocA^-$, DH701), which was transformed with plasmids (see **Supplementary Table 3**) encoding the indicated FocA variants. The dotted horizontal line indicates the threshold β-galactosidase activity (in Miller units), which equates to formate in a strain lacking FocA (first histogram on the left). β-galactosidase enzyme activity below the dotted line, and represented by the green bar, equating to reduced intracellular formate levels in the strain producing wild-type FocA (see second histogram from left), indicating formate efflux. Histograms with β-galactosidase activity above the dotted line indicate intracellular accumulation of formate, equating to impaired efflux. **C** Formate uptake was analyzed in the *focA* and *pflA* double-null mutant ($FocA^-$, inactive PflB [PflBin]), DH601, which cannot make formate intracellularly and therefore was cultivated in the presence of 20 mM sodium formate provided exogenously (see Methods). In this case, impaired FocA-dependent import of formate (indirectly measured as β-galactosidase enzyme activity) delivers a threshold depicted by the light blue bar (app. 1000 Miller units; histogram on the left). Restoration of wild-type FocA on a plasmid into the strain increases β-galactosidase activity to app. 1500 Miller units (second histogram from left). Thus, removal of the central 20 amino acids of the N-terminal domain (ΔL11-P31), or exchanging G23 for phenylalanine, inactivates the protein. All experiments were performed in triplicate with three biological replicates. Data are presented as mean values +/- SEM mean. n.d., not determined.

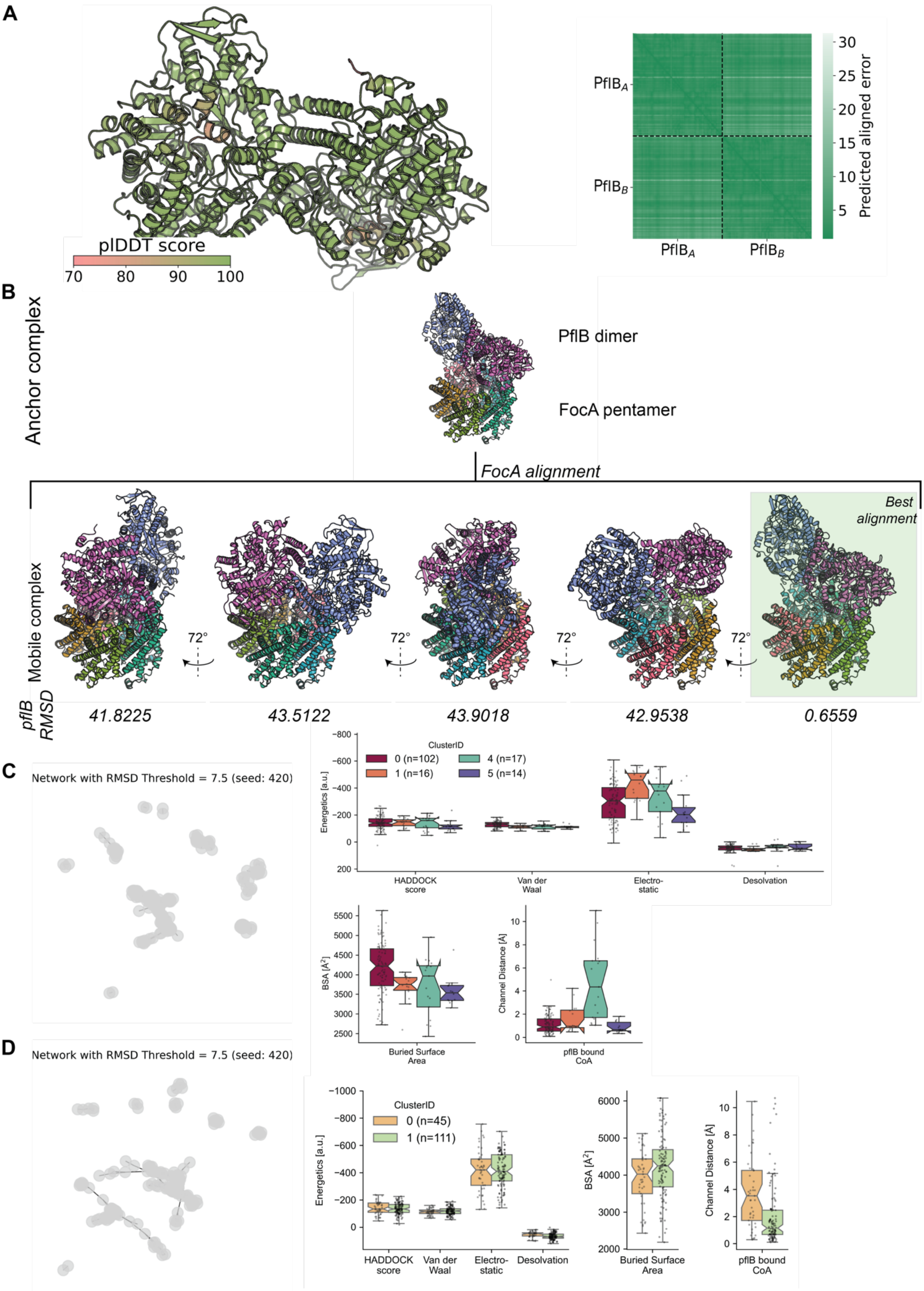

Supplementary Figure 21. Docking and angular rotation sampling implemented for the FocA-PflB docking calculations. (A) AlphaFold 2 result of the PflB structure prediction. The model is of high confidence, both obvious in the pIDDT and PAE scoring. (B) Angular rotation sampling

workflow. (C) Clustering of FocA full-length – PflB docking. (D) Clustering of FocA deltaN30 – PflB docking. Boxplots are overlaid by the datapoints as scatterplot. The box minima represent the 25th percentile, the box maxima the 75th percentile, the Notch indicates the data's median, whiskers extend to the minimum and maximum values inside a 1.5 interquartile range. n refers to individual water-refined protein models generated in the HADDOCK workflow (200 models in total).

Uncropped Silver Stain
Supplementary Figure 1A

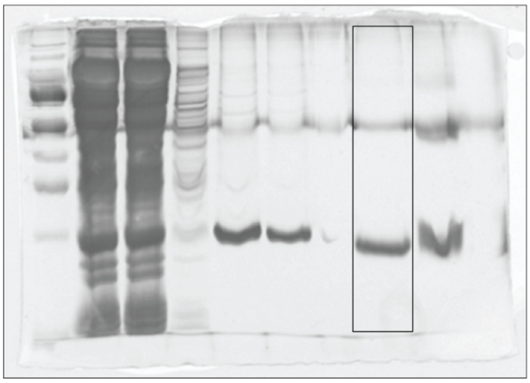

Uncropped BN-Page
Supplementary Figure 1B

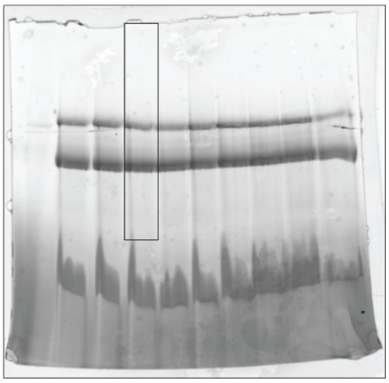

Supplementary Figure 22. Uncropped Gels. Uncropped gels and point of view (boxes), presented in Supplementary Figure 1.

Uncropped Silver Stain

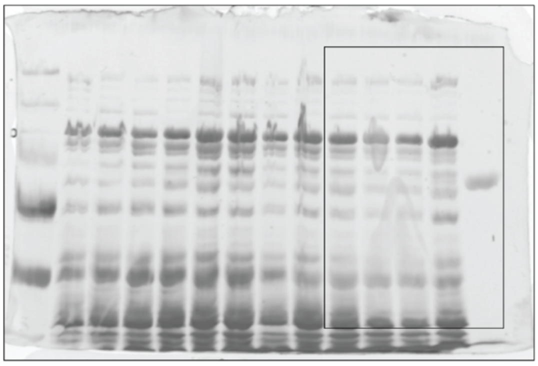

Uncropped Western Blot

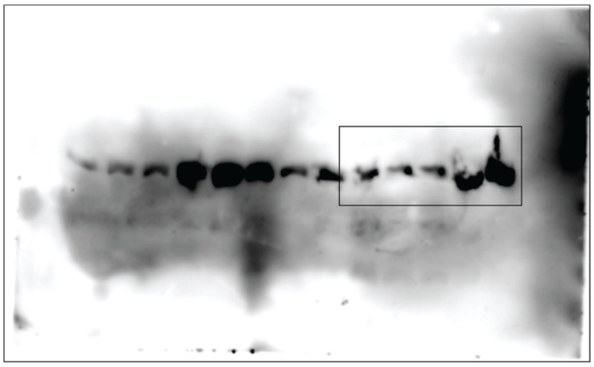

Supplementary Figure 23. Uncropped Blots. Uncropped blots and point of view (boxes), presented in Supplementary Figure 12.

Supplementary Table 1: Intermolecular cross-links between FocA variants and full-length PflB. The amino acids cross-linked are shown in bold and are underlined. Exemplary mass spectra are shown in **Fig. S19**. Note that in peptide FocA3–17, the cross-link site cannot be distinguished between D4 and D8.

	**FocA variant**	**Cross-linker**	**Measured *m/z***	**$[M+H]^+_{theo.}$**	**Charge state**	**Mass deviation [ppm]**	**FocA cross-linking site**	**PflB cross-linking site**
**A**	FocA	sulfo-SDA	949.922	1898.833	+2	1.8	^{231}FWTAVGSAP**E**^{240}	^{321}TPE**Y**DE^{326}
**B**	FocA	sulfo-SDA	1481.750	2962.494	+2	-0.11	^{3}A**D**NPF**D**LLLPAAMAK^{17}	^{50}ATTTLWD**K**VME^{60}
**C**	FocA	sulfo-SDA	1174.242	3520.709	+3	0.76	^{3}A**D**NPF**D**LLLPAAMAK^{17}	^{8}LATAWEGFT**K**GDWQNE^{23}
**D**	FocA	sulfo-SDA	1322.009	3964.005	+3	1.57	^{3}A**D**NPF**D**LLLPAAMAK^{17}	^{662}DGISYTFSIVPNALG**K**DDEVR^{682}
**E**	$FocA_{1-32}$	DSBU	474.289	1420.852	+3	-0.41	^{22}AGVY**K**ATK^{29}	^{454}**K**LK^{456}
**F**	$FocA_{1-32}$	DSBU	399.983	1596.911	+4	-0.4	^{22}AGVY**K**ATK^{29}	^{386}KFAAK^{390}
**G**	$FocA_{1-32}$	DSBU	738.699	2214.083	+3	0.32	^{22}AGVY**K**ATK^{29}	^{14}GFT**K**GDWQNE^{23}
**H**	$FocA_{1-32}$	DSBU	606.591	2423.341	+4	0.78	^{27}AT**K**HPLK^{33}	^{162}K**S**GVLTGLPDAYGR^{175}
**I**	$FocA_{1-32}$	DSBU	617.340	2466.335	+4	0.6	^{22}AGV**Y**KATK^{29}	^{162}**K**SGVLTGLPDAYGR^{175}

Supplementary Table 2 Oligonucleotide primers used for site directed mutagenesis.

Primers	Sequence 5' → 3'[a]
focA_E21A_fw	CAAAGTGGCCGAAGCGGCGGGTGTC
focA_E21A_rev	GACACCCGCCGCTTCGGCCACTTTG
focA_G23F_fw	GCCGAAGAGGCGTTTGTCTATAAAGC
focA_G23F_rev	GCTTTATAGACAAACGCCTCTTCGGC
focA_Y25A_fw	GAGGCGGGTGTCGCTAAAGCAACGAAAC
focA_Y25A_rev	GTTTCGTTGCTTTAGCGACACCCGCCTC
focA_K26A_fw	GAGGCGGGTGTCTATGCGGCAACGAAACATCC
focA_K26A_rev	GGATGTTTCGTTGCCGCATAGACACCCGCCTC
focA_K26E_fw	AGGCGGGTGTCTATGAAGCAACG
focA_K26E_rev	CTTCGGCCACTTTGGCCATTG
focA_T28A_fw	GTCTATAAAGCAGCGAAACATCCGC
focA_T28A_rev	GCGGATGTTTCGCTGCTTTATAGAC
focA_K29A_fw	CTATAAAGCAACGGCACATCCGCTTAAG
focA_K29A_rev	CTTAAGCGGATGTGCCGTTGCTTTATAG
focA_K29E_fw	TAAAGCAACGGAACATCCGCTTAAG
focA_K29E_rev	TAGACACCCGCCTCTTCG

[a]Underlined bases highlight the substitution introduced

Supplementary Table 3 Strains and plasmids used in this study

Strains, plasmids, phage	Relevant genotype or characteristics	Reference or source
Strains		
BL21 (DE3)	F[-] *ompT hsdS*(rB- mB-) *gal dcm lacY1* (DE3)	Invitrogen, Carlsbad, USA
DH4100	MC4100 *λ(fdhF::lacZ)*	[6]
DH4200	Like DH4100, but *focA* codon 209 changed to AAC	[7]
DH4300	Like DH4100, but *focA* codon 91 changed to GCT	[8]
DH701	MC4100 *focA*[-] *λ(fdhF$_P$::lacZ)*	[6]
DH601	MC4100 *focA*[-] Δ*pflA* Ω*(pflA::cat* pACYC184*) λ(fdhF$_P$::lacZ)*	[9]
Plasmids		
pCA24NpflB	Cm[r], pCA24N vector encoding the *pflB gene* with a *N*-terminal His-tag	National BioResources Project (NIG, Japan)
pfocA3	Amp[r], pASK-IBA3focA expression vector with the *focA* gene encoding *C*-terminally StrepII-tagged FocA	[10]
pfocAH209N	pASK-IBA3focA expression vector carrying the *focA* gene derivative encoding *C*-terminally StrepII-tagged FocA-H209N	[7]
pfocA ΔL11-P31	pASK-IBA3focA expression vector with the *focA* gene with a deletion of leucine 11 to proline 31	[9]
pfocA E21A	pASK-IBA3focA expression vector with the *focA* gene encoding the amino acid exchange glutamate 21 to alanine	This study
pfocA G23F	pASK-IBA3focA expression vector with the *focA* gene encoding the amino acid exchange glycine 23 to phenylalanine	This study
pfocA Y25A	pASK-IBA3focA expression vector with the *focA* gene encoding the amino acid exchange tyrosine 25 to alanine	This study
pfocA K26A	pASK-IBA3focA expression vector with the *focA* gene encoding the amino acid exchange lysine 26 to alanine	This study
pfocA K26E	pASK-IBA3focA expression vector with the *focA* gene encoding the amino acid exchange lysine 26 to gutamate	This study
pfocA T28A	pASK-IBA3focA expression vector with the *focA* gene encoding the amino acid exchange threonine 28 to alanine	This study
pfocA K29A	pASK-IBA3focA expression vector with the *focA* gene encoding the amino acid exchange lysine 29 to alanine	This study

pfocA K29E	pASK-IBA3focA expression vector with the *focA* gene encoding the amino acid exchange lysine 29 to glutamate	This study

Supplementary Table 4: Distance restraints. Cross-linking restraints used for docking. d^0 defines the target distance, whereas d^- and d^+ defines the lower and upper margin, respectively. For FocAΔN30, residues below 30 were re-defined to residue 30 implicitly to include these cross-links in docking.

Residue1	Protein1	Residue2	Protein2	d^0	d^-	d^+
17	FocA	312	PflB	30	30	0
26	FocA	197	PflB	30	30	0
17	FocA	32	PflB	30	30	0
17	FocA	441	PflB	30	30	0
112	FocA	463	PflB	30	30	0
17	FocA	126	PflB	30	30	0
191	FocA	298	PflB	30	30	0
17	FocA	338	PflB	30	30	0
17	FocA	192	PflB	30	30	0
17	FocA	643	PflB	30	30	0
187	FocA	136	PflB	30	30	0
28	FocA	96	PflB	30	30	0
26	FocA	726	PflB	30	30	0
29	FocA	395	PflB	30	30	0
240	FocA	324	PflB	30	30	0
4	FocA	56	PflB	30	30	0
4	FocA	17	PflB	30	30	0
4	FocA	677	PflB	30	30	0
26	FocA	454	PflB	30	30	0
26	FocA	386	PflB	30	30	0
26	FocA	17	PflB	30	30	0
29	FocA	163	PflB	30	30	0
25	FocA	162	PflB	30	30	0

Supplementary References

1 Wang, Y. *et al.* Structure of the formate transporter FocA reveals a pentameric aquaporin-like channel. *Nature* **462**, 467–472 (2009). https://doi.org/10.1038/nature08610

2 Waight, A. B., Love, J. & Wang, D. N. Structure and mechanism of a pentameric formate channel. *Nat Struct Mol Biol* **17**, 31–37 (2010). https://doi.org/10.1038/nsmb.1740

3 Lu, W. *et al.* pH-dependent gating in a FocA formate channel. *Science* **332**, 352–354 (2011). https://doi.org/10.1126/science.1199098

4 Czyzewski, B. K. & Wang, D. N. Identification and characterization of a bacterial hydrosulphide ion channel. *Nature* **483**, 494–497 (2012). https://doi.org/10.1038/nature10881

5 Lu, W. *et al.* Structural and functional characterization of the nitrite channel NirC from Salmonella typhimurium. *Proc Natl Acad Sci U S A* **109**, 18395–18400 (2012). https://doi.org/10.1073/pnas.1210793109

6 Hunger, D., Doberenz, C. & Sawers, R. G. Identification of key residues in the formate channel FocA that control import and export of formate. *Biol Chem* **395**, 813–825 (2014). https://doi.org/10.1515/hsz-2014-0154

7 Kammel, M., Trebbin, O., Pinske, C. & Sawers, R. G. A single amino acid exchange converts FocA into a unidirectional efflux channel for formate. *Microbiology (Reading)* **168** (2022). https://doi.org/10.1099/mic.0.001132

8 Kammel, M. & Sawers, R. G. The FocA channel functions to maintain intracellular formate homeostasis during Escherichia coli fermentation. *Microbiology (Reading)* **168** (2022). https://doi.org/10.1099/mic.0.001168

9 Kammel, M., Hunger, D. & Sawers, R. G. The soluble cytoplasmic N-terminal domain of the FocA channel gates bidirectional formate translocation. *Mol Microbiol* **115**, 758–773 (2021). https://doi.org/10.1111/mmi.14641

10 Falke, D. *et al.* Unexpected oligomeric structure of the FocA formate channel of Escherichia coli : a paradigm for the formate-nitrite transporter family of integral membrane proteins. *FEMS Microbiol Lett* **303**, 69–75 (2010). https://doi.org/10.1111/j.1574-6968.2009.01862.x
