# Supplementary material for: Conserved hydrophilic checkpoints tune FocA-mediated formate:H+ symport
Source: Nat Commun. 2025 Oct 27;16:9476. doi: 10.1038/s41467-025-65159-3 (PMC12559229; doi:10.1038/s41467-025-65159-3)
Supplement: Supplementary file 2 — Description of Additional Supplementary Files [file 41467_2025_65159_MOESM2_ESM.pdf]

Description of Additional Supplementary Files

File Name: Supplementary Movie 1
Description: 3D variability of wild-type FocA. The protein is displayed as a cartoon representation, with the cryo-EM density shown as a transparent volumetric morph.
